# Mapping the local dielectric constant of a biological nanostructured system

**DOI:** 10.3762/bjnano.12.11

**Published:** 2021-01-28

**Authors:** Wescley Walison Valeriano, Rodrigo Ribeiro Andrade, Juan Pablo Vasco, Angelo Malachias, Bernardo Ruegger Almeida Neves, Paulo Sergio Soares Guimarães, Wagner Nunes Rodrigues

**Affiliations:** 1 Departamento de Física, ICEx, Universidade Federal de Minas Gerais, Av. Antônio Carlos 6627, 31270-901 Belo Horizonte, Minas Gerais, Brazil; 2 Centro de Microscopia, Universidade Federal de Minas Gerais, Av. Antônio Carlos 6627, 31270-901 Belo Horizonte, Minas Gerais, Brazil; 3 Institute of Theoretical Physics, École Polytechnique Fédérale de Lausanne EPFL, CH-1015 Lausanne, Switzerland

**Keywords:** dielectric constant, electrostatic force microscopy (EFM), natural photonic crystals, relative permittivity, structural colors

## Abstract

The aim of this work is to determine the varying dielectric constant of a biological nanostructured system via electrostatic force microscopy (EFM) and to show how this method is useful to study natural photonic crystals. We mapped the dielectric constant of the cross section of the posterior wing of the damselfly *Chalcopteryx rutilans* with nanometric resolution. We obtained structural information on its constitutive nanolayers and the absolute values of their dielectric constant. By relating the measured profile of the static dielectric constant to the profile of the refractive index in the visible range, combined with optical reflectance measurements and simulation, we were able to describe the origin of the strongly iridescent wing colors of this Amazonian rainforest damselfly. The method we demonstrate here should be useful for the study of other biological nanostructured systems.

## Introduction

The dielectric constant, or relative permittivity, is a fundamental physical property that is crucial for describing various biological, chemical, or physical phenomena. It is a material property associated to the decrease of the electric force between two point charges due to the medium. Therefore, it modulates the interaction between charged particles within materials and also the interaction of electromagnetic radiation with matter. Accordingly, it plays a fundamental role in fields such as the full understanding of proteins [1–2] or in the development of solar cells [3].

Natural photonic crystals are exciting nanostructured systems in which the dielectric properties play a fundamental role [4]. Many of them are biological systems where the richness of colors, produced by different strategies found in nature, is astonishing [5–6]. Studies of the origin of physical colors in insects are numerous in the literature and the most commonly used tools are non-local optical reflectance, electron microscopy, and scanning probe microscopy techniques, which give support to theoretical models aiming to describe the measured optical properties [7]. However, all these techniques directly reveal only the structure with nanometric resolution, the local dielectric response is indirectly inferred from a model [8–10]. Despite the large number of studies, the local dielectric properties of natural photonic crystals remain essentially undetermined due to the great difficulties in measuring the dielectric response at the nanometric scale [11]. The nanometric local relative permittivity of a natural photonic crystal has not been directly measured yet.

Fumagalli et al. [12–15], and Riedel et al. [16] developed several techniques of electrostatic force microscopy (EFM) to extract the relative permittivity at the nanoscale, allowing for new fields to be explored. Here we use EFM to map the relative permittivity of nanostructures within the wings of the *Chalcopteryx rutilans* damselfly [17–19]; nanostructures which make it a natural photonic crystal. We obtain quantitative information about the wing structure and its local relative permittivity values. We also simulate the optical reflectance using the extracted spatial profile of the relative permittivity and compare it with the measured reflectance in the visible range, obtaining a good correlation. In this way, we can provide a full description of the origin of the shimmering colors of the posterior wings of the *Chalcopteryx rutilans* damselfly male. This technique should be useful in the study of similar systems enhancing the investigation possibilities of natural photonic crystals.

## Results and Discussion

In damselflies, color has many functions, the most important being sex recognition, courtship, and territory defense behavior [19]. In *Chalcopteryx rutilans* – a damselfly found in the Amazonian rain forest – those functions are performed by the male by displaying its strongly iridescent hind wings. The phenomenon of iridescence results from both diffraction and interference in the damselfly wings, and all observed colors result from a multilayer structure, that is, these wings are natural one-dimensional photonic crystals [7–10].

**Figure 1:**
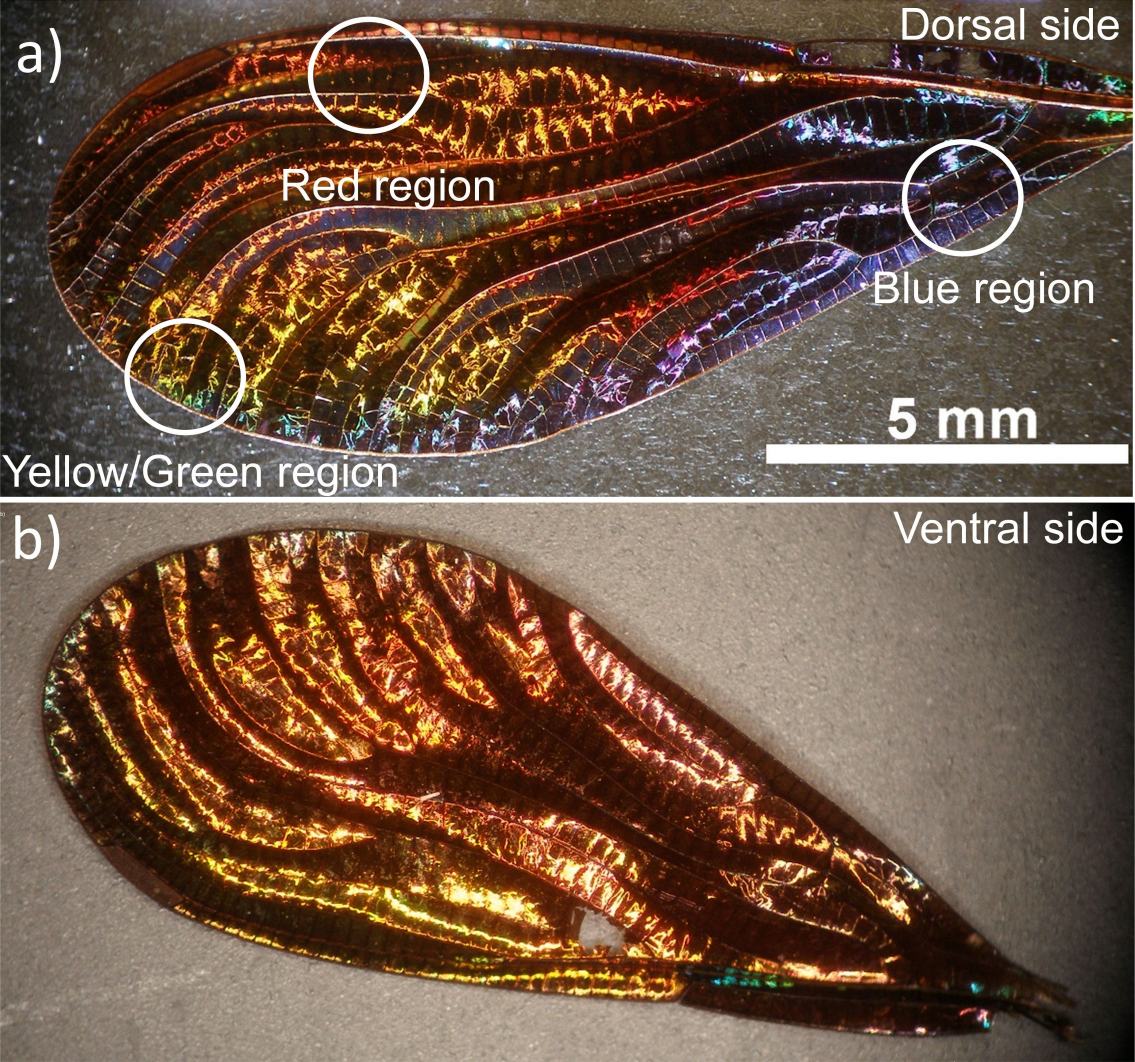
Optical images of the iridescent hind wing of the male damselfly *Chalcopteryx rutilans* (Rambur) (Odonata, Polythoridae). The dorsal side (a) displays colors that span all the visible wavelength spectrum. The image in (b) shows the ventral side, which is almost all red, remarkably similar to the iridescent wing of the female *C. rutilans*.

For our measurements, we chose three different color regions of the iridescent posterior wings of the male *Chalcopteryx rutilans* to study, that is, the yellow/green, red, and blue regions observed at the dorsal side, Figure 1a. The ventral side shows several shades of red, Figure 1b.

**Figure 2:**
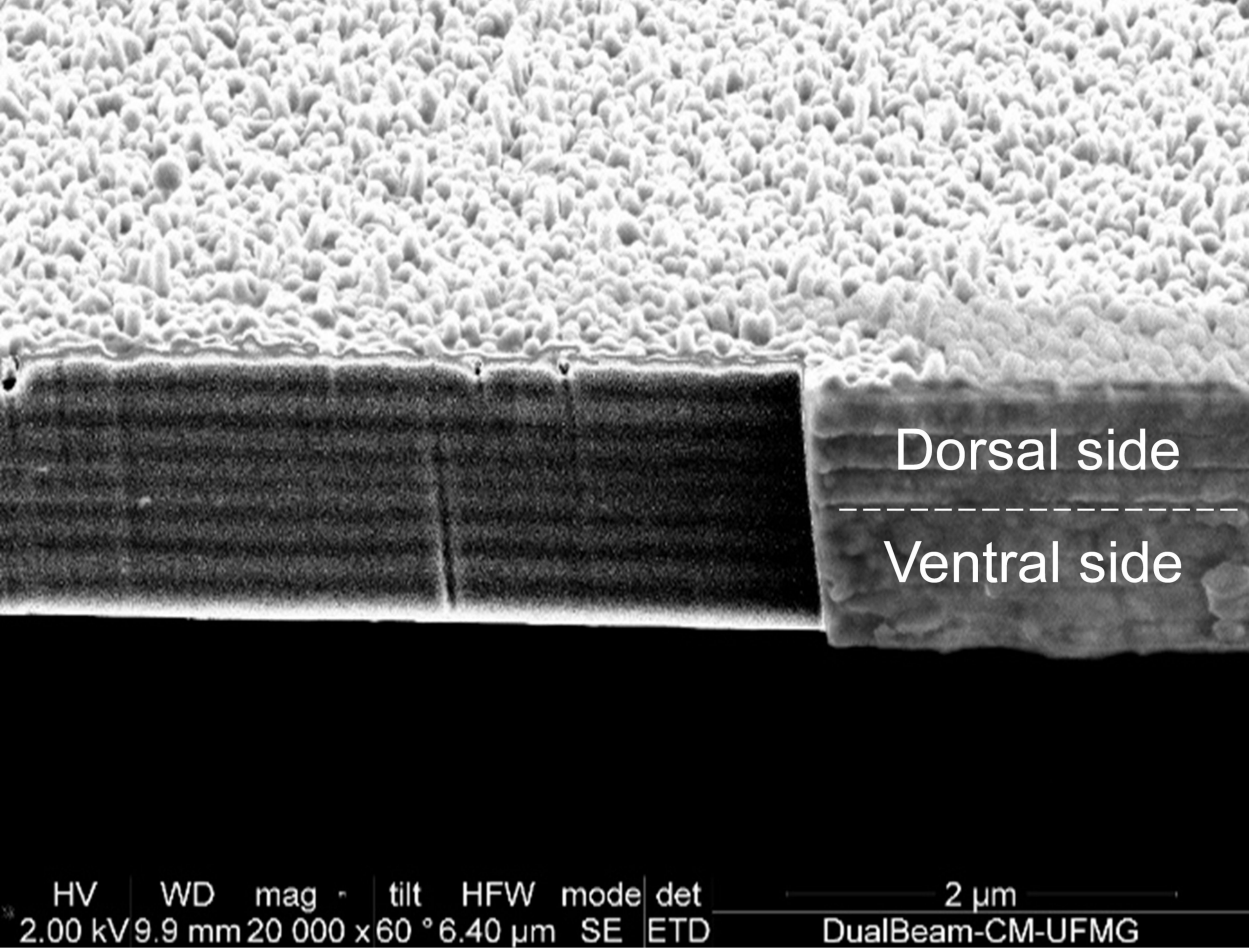
SEM image of the cross section of a red region fragment of the *Chalcopterix rutilans* male rear wing. The smooth region was obtained by polishing using FIB.

The scanning electron microscopy (SEM) image presented in Figure 2 shows the nanostructured section of a fragment of the red region indicated in Figure 1a. The section was partially polished using a focused ion beam (FIB) and the multilayered structure is clearly visible. The corrugated surface is the wax layer that covers the damselfly wings [20]*.* Detailed electron microscopy and mass spectrometry studies of this natural nanostructured system can be found in Valeriano [17] and Carr et al. [18].

### Relative permittivity determination via EFM

The EFM measurements were performed in the conventional double-pass mode, which means that the probe executes two scans. The first scan measures the sample topography in tapping mode and the second scan mimics the profile at a defined lift height *Z*_lift_ applying a voltage *V*_DC_ between the tip and the conductive substrate [21].

The tip is mechanically forced to oscillate, during the second pass, at the resonance frequency of the cantilever, *f*_0_. Variations in the local relative permittivity properties of the sample will lead to different tip–sample force gradients, which promote a shift Δ*f*_0_ in the tip oscillation frequency [21–22] which is, approximately,

[1]
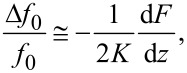


where d*F/*d*z* is the tip–sample force gradient and *K* is the spring constant of the cantilever.

The tip–sample-substrate system constitutes a capacitor with the sample (wing) as part of the relative permittivity region, so the force between tip and substrate can be modeled as

[2]
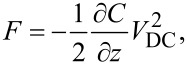


where *C* is the system capacitance and *V*_DC_ is the applied tip–sample bias.

From Equation 1 and Equation 2 we have:

[3]
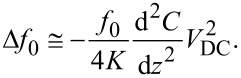


This equation relates the frequency shift Δ*f*_0_ to the applied bias voltage *V*_DC_ and is the measured EFM signal. The bias-independent term in Equation 3 is defined as α, given by

[4]
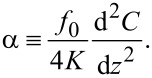


Since *f*_0_, *K* and *V*_DC_ are well determined, local variations of the measured frequency shift Δ*f*_0_ are associated with changes in the second derivative of the capacitance in Equation 3 and Equation 4. The capacitance depends both on the geometry and on the relative permittivity of the medium. Hence, we only need to use a suitable capacitance model to be able to determine the local relative permittivity of the sample from the EFM data.

The capacitance model applied here considers a conical tip and an infinite flat surface with relative permittivity ε_r_ and thickness *h*, which has a capacitance expressed as

[5]
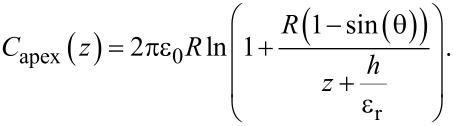


**Figure 3:**
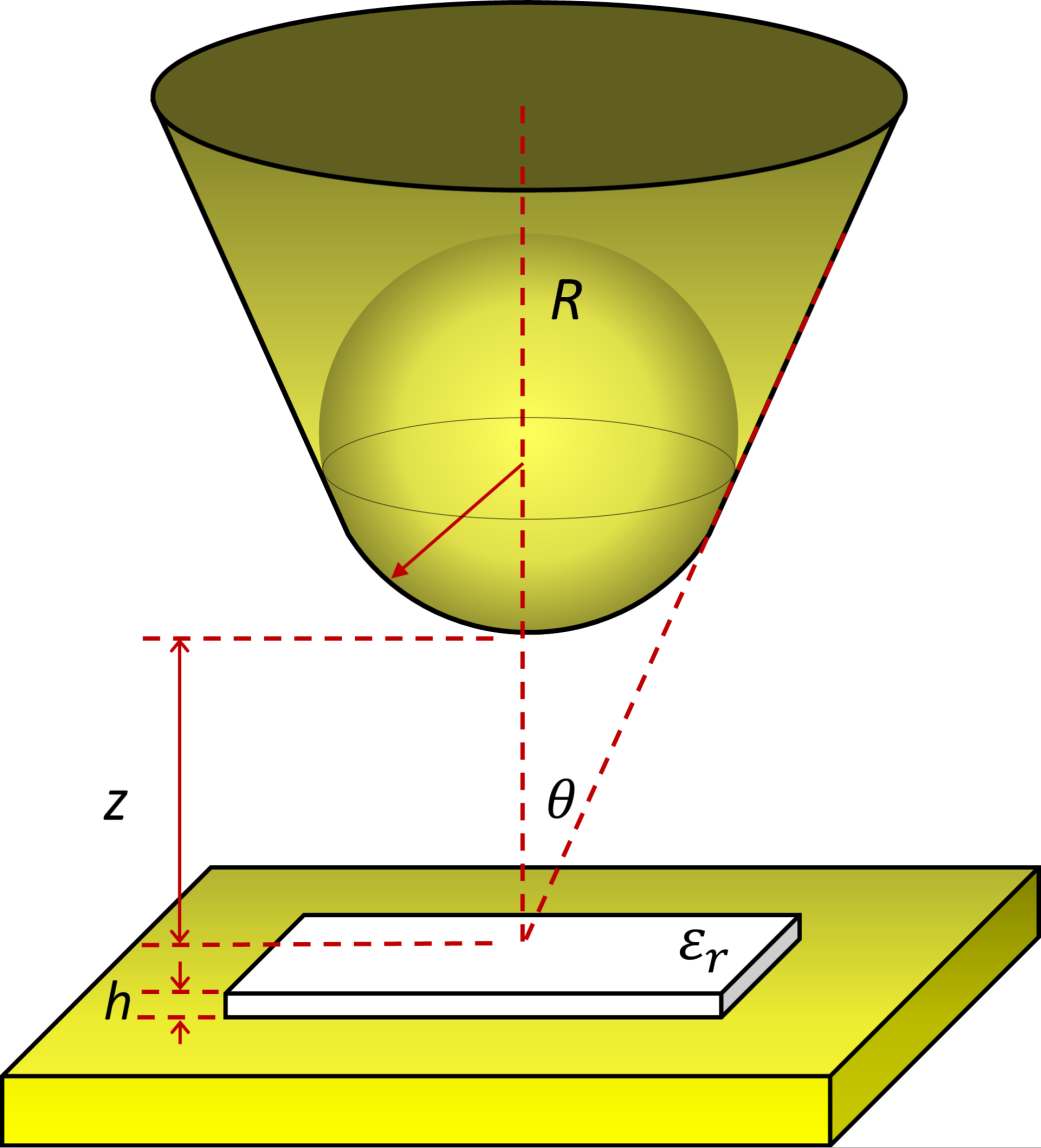
Capacitance model with tip, sample, conductive plate, and the parameters used in our calculations. *R* is the tip apex radius, θ is the tip conical angle, *z* is the tip–sample distance, *h* is the sample thickness and ε_r_ is the relative permittivity of the sample. The sample is shown on top of the conductive substrate (yellow plate), which constitutes the bottom plate of the capacitor.

Inserting Equation 5 into Equation 4 leads to an expression from which the relative permittivity ε_r_ can be extracted:

[6]
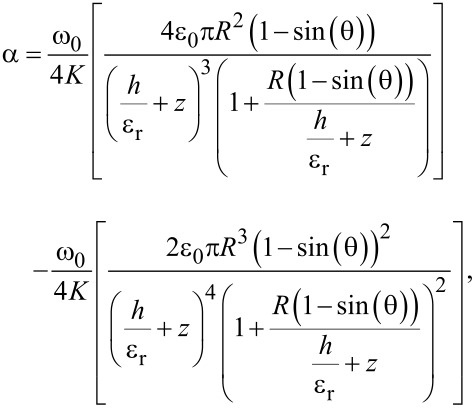


where *R* is the tip apex radius, θ is the tip conical angle, *z* is the tip–sample distance, and *h* is the sample thickness. Figure 3 shows a scheme of the model, which is commonly applied for such a configuration [12–16].

This capacitance model is valid under the following conditions: i) tip–sample distance *z* between 0 and 100 nm; ii) maximum sample thickness *h* around 100 nm; iii) relative permittivity ε_r_ smaller than 100; iv) nominal tip radius *R* between 30 and 200 nm; v) tip cone angle θ between 10° and 45° [12].

### Determination of the coefficient α

**Figure 4:**
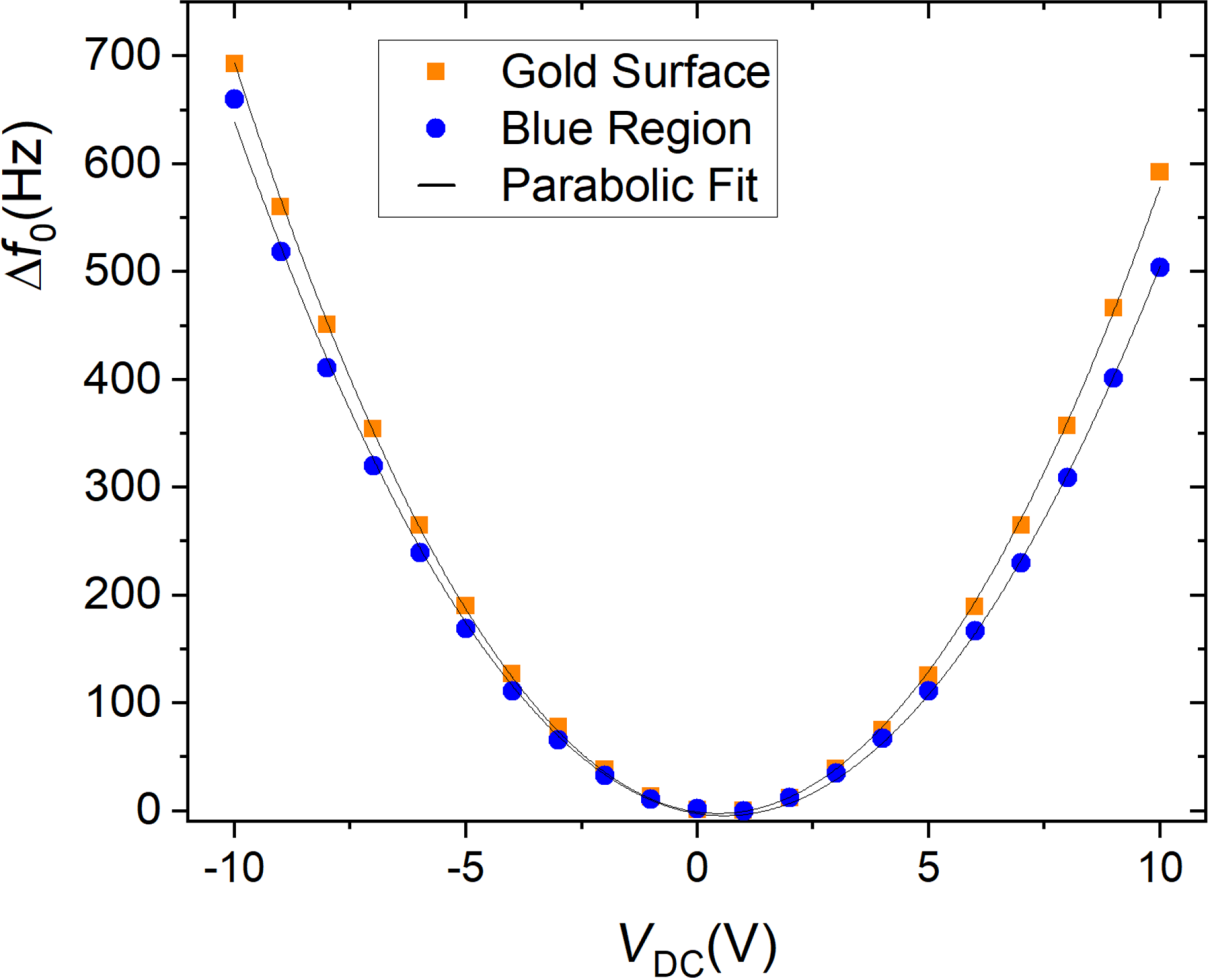
Frequency shift as a function of the bias voltage for the gold surface that is the conductive substrate in Figure 3, (orange squares) and for the blue region of the wing (blue circles). The fit using Equation 7 results in α(gold) = (6.37 ± 0.03) Hz/V^2^, and α(blue region) = (5.75 ± 0.04) Hz/V^2^.

For the experiments we use an atomic force microscope in the EFM mode, which measures the frequency shift for each bias voltage at each position of the sample. We varied the bias voltage from −10 V to +10 V, in steps of 1 V. Plotting the frequency shift as a function of the bias voltage, we obtain a parabolic function, as can be seen in Figure 4. Fitting the data with the function

[7]
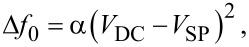


where *V*_SP_ is the tip–sample surface potential difference due to their different work functions [21], we obtain the coefficient α.

### Construction of the relative permittivity map

**Figure 5:**
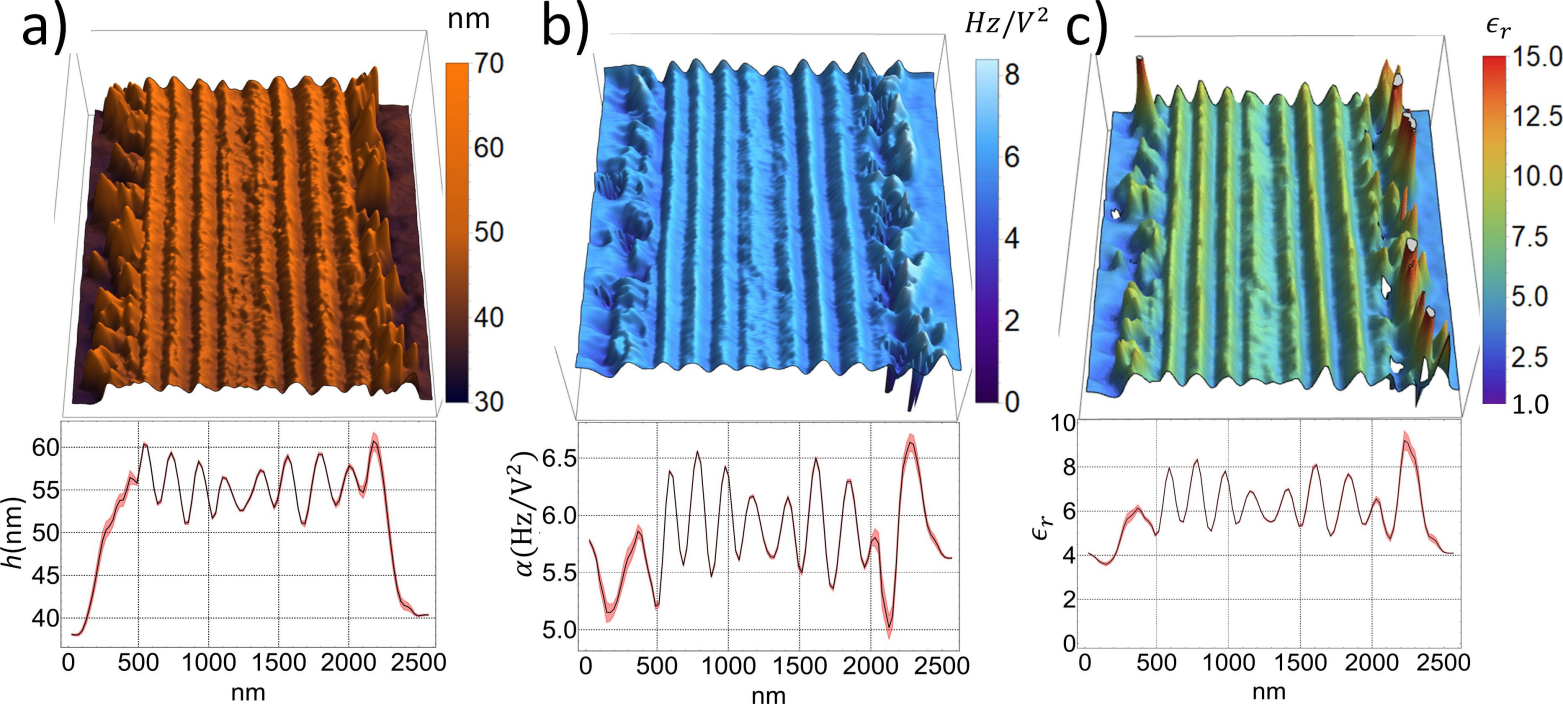
(a) Topographic map; (b) α coefficient map; and (c) relative permittivity map. The average of all line profiles is shown below each image. All maps refer to the same red colored wing region.

From the topographic image, the local thickness of the sample *h* is determined for each pixel, as we can see in Figure 5a.

EFM measurements were carried out in the same sample region, varying the bias voltage from −10 V to +10 V. This resulted in 21 images, each one an array of frequency shift values. For the same pixel element in the sample image, we have 21 pairs of values, that is, a frequency shift and its respective bias voltage. Through Equation 7 we obtain the α coefficient for each pixel element in the sample, Figure 5b.

**Figure 6:**
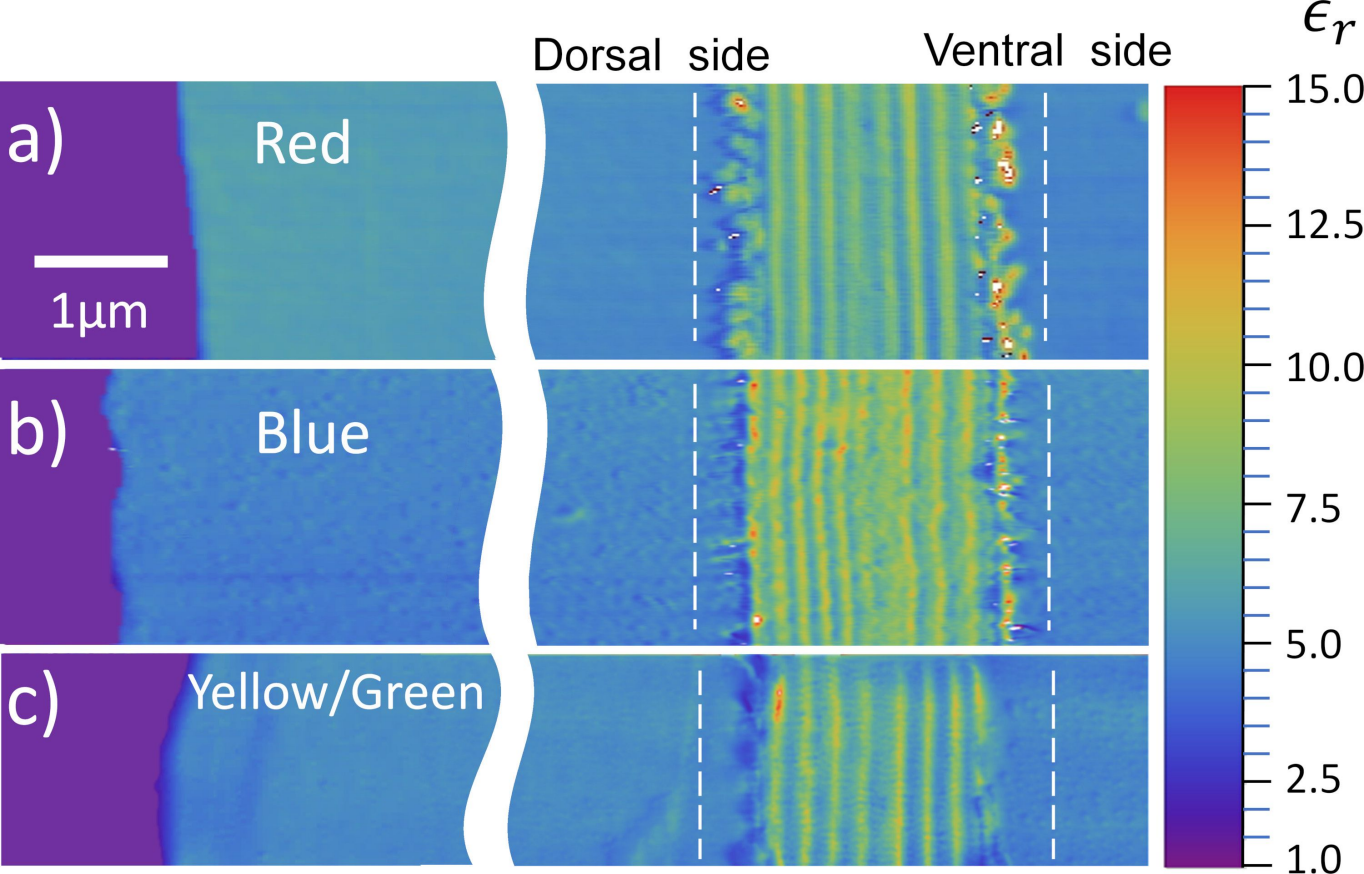
Relative permittivity image of three color regions of the hind wings of *Chalcopterix rutilans*: (a) red region, (b) blue region, and (c) yellow/green region. The color scale on the right side gives the values of the relative permittivity. On the left side, colored in purple, we have the Au/Cr surface. The areas that appear bluish in the images (ε_r_ around 4) correspond to the polymerized resin wrapping the wing. The wing slice lies between the white vertical dashed lines, which indicate the region where the profiles presented below in Figure 7 were measured.

The parameters in Equation 6, except the α coefficient and the thickness *h*, are the same for all pixel elements. Thus, solving the equation regarding the relative permittivity of each pixel, we construct a relative permittivity map as can be seen in Figure 5c and below in Figure 6. In the topographic map and the average profile, the different layers and their widths can be identified (Figure 5a). The α coefficient map and its average profile differ slightly from the topographic information, but some correspondences are identified. Thicker regions have a smaller α coefficient, as can be seen at the wing–resin interface (Figure 5b). There seems to be a correlation between topography (Figure 5a) and the relative permittivity ε_r_ (Figure 5c): the lower the topography, the larger the ε_r_. However, we observe a small ε_r_ value in the lower topography regions adjacent to the wing slab. Hence, topography crosstalk is small.

### *Chalcopterix rutilans* damselfly wings

Using the protocol described above, we constructed relative permittivity maps of the three color regions: red region (Figure 6a), blue region (Figure 6b), and yellow/green region (Figure 6c). The parameters used for the red region are *f*_0_ = 61.106 kHz, *K* = 1.19 N/m and *R* = (34.8 ± 0.2) nm; for the blue region they are *f*_0_ = 62.111 kHz, *K* = 1.87 N/m and *R* = (42.6 ± 0.2) nm; and for the yellow/green region the parameters are *f*_0_ = 67.972 kHz, *K* = 2.18 N/m and *R* = (35.9 ± 0.2) nm.

Similar to the case of Al_2_O_3_ (see Experimental section), the substrate region was set to ε_r_ = 1 since in this region there is only air between the probe and the gold substrate [15]. The relative permittivity of the polymerized resin in which the wing is embedded (see Experimental section) is ε_r_(resin) ≈ 4. The cross-sectional cut of the wing lies between the dashed lines, where the nanometric layers of the wing can be seen. The wax layers that cover both sides of the wings appear as the external discontinuous regions of the multilayered structure. The number of nanolayers and their thickness values change from one color region to another.

The ventral and dorsal sides shown in Figure 1 can be seen in cross section in the images of Figure 6, where the ventral side is shown on the right, and the dorsal side on the left. On the left side of the red region, the blue region, and the yellow/green region, the width of each nanolayer is (200 ± 9) nm, (150 ± 5) nm, and (185 ± 11) nm, respectively, see Figure 2 and Figure 5a. In all regions, the layers on the right side are about (210 ± 10) nm wide, matching the reddish color of the whole ventral side of the wing, as seen in Figure 1b.

**Figure 7:**
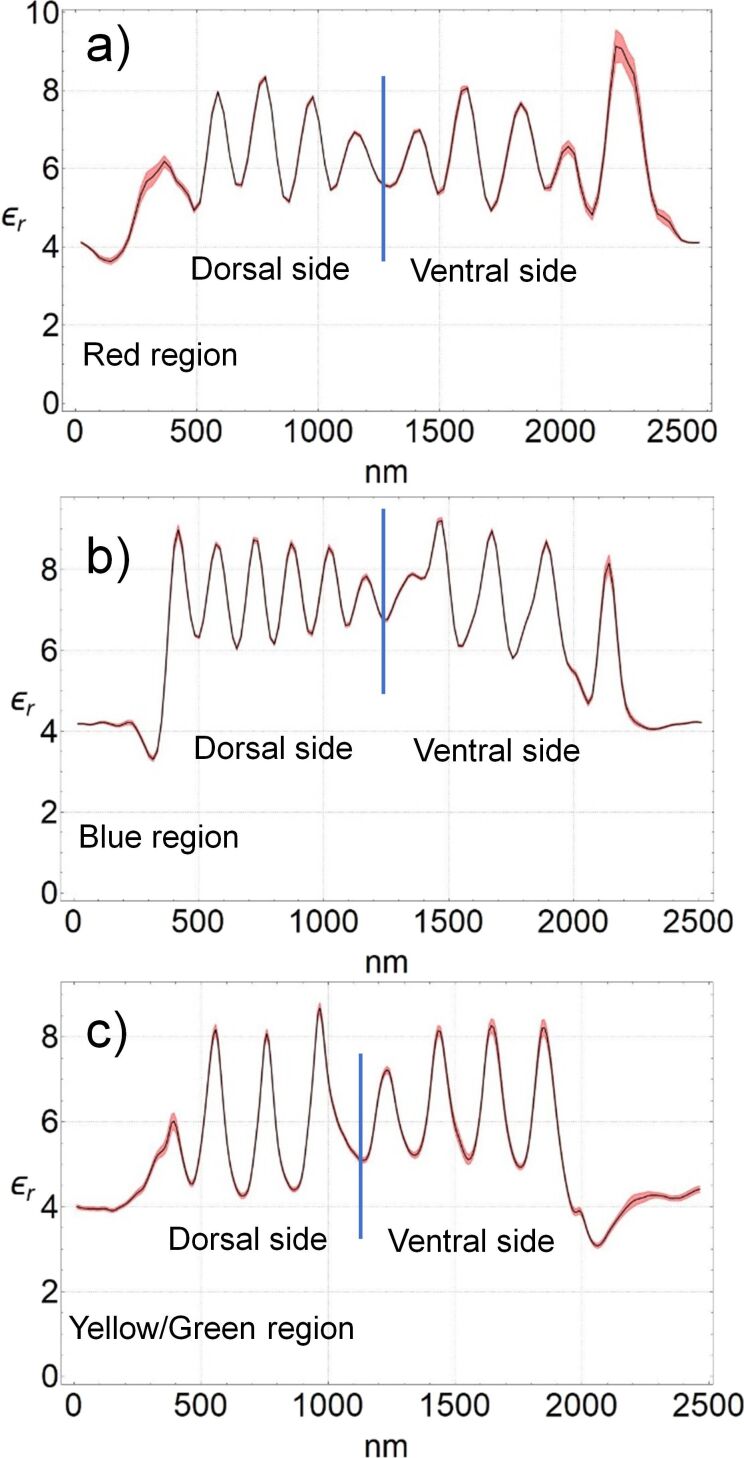
The black lines show the average profile of the relative permittivity of the cross sections of the wing between the dashed lines in Figure 6. These average profiles were obtained by averaging all the 128 profiles that constitute each map shown in Figure 6, that is, in (a) the red region, in (b) the blue region, and in (c) the yellow/green region. The peaks and valleys correspond to the nanometric layers that constitute the wing. The dielectric constant of the peaks ranges between 8 and 9, and that of the valleys between 5 and 6. The red shadow on the black lines shows the standard deviation.

Figure 7 shows the average value of the relativity permittivity, for each region, along the cross section of the wing (the area between the vertical white dashed lines in the figure). The values shown are obtained by averaging all of the profiles that constitute each of the relative permittivity maps of Figure 6. The peaks and valleys indicate the modulation of the relative permittivity of the different constituent layers of the wing.

During the preparation of the wing samples, there is a variation of about 10 nm in the thicknesses of the slices. This variation has not impacted the results, as can be seen in Figure 7, which demonstrates the reliability of this technique for thin samples.

The relative permittivity of the layers ranges from 6 ± 1 to 8 ± 1. The main differences between the regions are the thickness and the number of layers. Each multilayered structure is wrapped by a wax layer, which is the irregular region at the boundary of the wings in the maps of Figure 6, and as a valley in the relative permittivity profile with ε_r_(wax) ≈ 4 in Figure 7.

Using time-of-flight secondary ion mass spectrometry (TOF-SIMS), Carr et al. [18] concluded that the wing layers consist of mostly chitin with an alternating content of melanin. Chitin forms the structure and melanin modulates the relative permittivity along the cross section. From the results shown in Figure 7, we can see that, in addition, the number of layers and their thickness varies from one color region to the other. Comparing the composition of the layers measured in the TOF-SIMS study [18] with the relative permittivity maps of this work, we can say that melanin-rich layers have a relative permittivity of 8 ± 1, while low melanin concentration layers exhibit a value of 6 ± 1.

### The structural color

As shown in Figure 1, the posterior wings of the male *Chalcopteryx rutilans* have a wide range of structural colors that covers almost the entire visible spectrum. We now seek to correlate the relative permittivity information obtained via EFM with the photonic behavior of the wing. The wing has several layers with thicknesses comparable to the wavelengths of visible light. Through refraction and diffraction, which depend on the thicknesses, refractive index, and the number of layers, light of certain wavelengths is reflected selectively with higher intensities, generating the observed colors. As shown in Figure 6 and Figure 7, the layers vary in quantity and thickness in each color region. The blue region exhibits the thinnest layers, the red region the thickest, and the yellow/green region has layers of intermediate thickness. It is interesting to note that for the red region the thickness of the layers is about the same at the dorsal and ventral sides of the wing, consistent with the fact that the ventral side (Figure 1b) only shows red shades.

The profile of the refractive index in each color region of the wing could, in principle, be directly obtained from the measured values of relative permittivity. However, the values obtained in the measurements of Figure 7 are those of the static dielectric constant, ε_r_(ω = 0), while for obtaining the values of the refractive index in the visible range one needs the values of the relative permittivity in the visible range, ε_r_(ω→∞). For solid-state cubic crystals, the two values are related by the Lyddane–Sachs–Teller relation [23], which gives the ratio ε_r_(0)/ε_r_(∞) in terms of the ratio between the squared values of the long-wavelength longitudinal and transverse optical phonons in the crystal. The Lyddane–Sachs–Teller relation has been extended to other crystalline systems and disordered materials [24–26] but its application for the present case, chitin with a varying concentration of melanin, is not straightforward. There is a direct relation between the refractive index and the measured relative permittivity. Therefore, to simulate the optical reflectance of the damselfly wing, we consider that the refractive index varies along the cross section of each region of the wing following the continuous profiles shown in Figure 7, treating the minimum and maximum values of each profile as fitting parameters. The continuous profiles are discretized in layers thin enough to give the smoothest possible variation and then the transfer matrix technique [27] is used to simulate the reflectance of the structure. This is actually a similar process as that used by Vukusic and Stavenga [7] and Stavenga et al. [8], except that in those works the spatial profile of the refractive index was taken to be proportional to the gray scale in TEM images. That is, it was assumed that the optical density is directly proportional to the electronic density. We consider our method to be more reliable since there is a direct relation between the relative permittivity and the refractive index.

**Figure 8:**
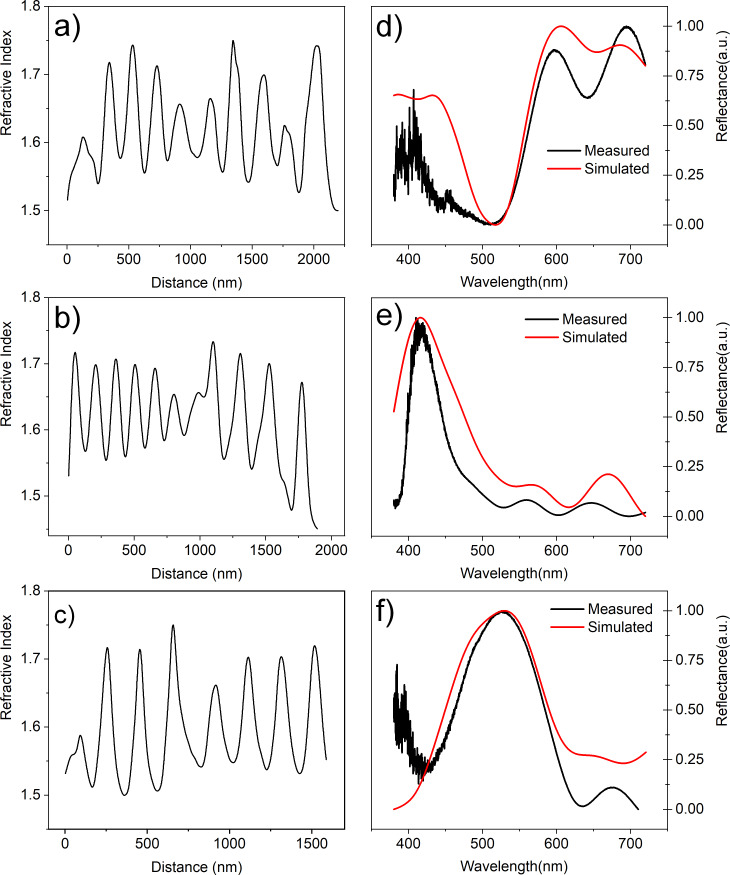
Panels on the left show the refractive index profile used in the simulation and those on the right the respective measured and simulated reflectance. In (a) and (d) we see the red region, in (b) and (e) the blue region, and in (c) and (f) the yellow/green region.

Figure 8 shows the results of the simulation. On the left panels, the profile of the refraction index used to fit the optical reflectance are shown for each color region of the wing. As explained above, these are the same profiles as obtained from the measurement of the relative permittivity, shown in Figure 7, only with the vertical scale changed for values of the refractive indexes to fit the reflectance measurements. The right panels show the respective measured reflectance values and the fits obtained with the refraction index profiles shown on the left.

According to the results shown in Figure 8, the refraction index along the cross section of the damselfly wings varies from 1.52 ± 0.02 to 1.72 ± 0.02. The layers are essentially composed of chitin with varying melanin concentration from layer to layer, as discussed above, and these values are in good agreement with other determinations of the refractive indexes of chitin and melanin [8–10,28].

## Conclusion

We have demonstrated that electrostatic force microscopy (EFM) is a reliable and useful tool to directly measure the relative permittivity of a natural photonic crystal. We showed how to obtain maps of the relative static permittivity with nanometric resolution, thus obtaining direct information about the internal structure of biological systems and their dielectric properties on the nanoscale. We applied the method to map the static relative permittivity of the cross section of the posterior wing of the Amazonian damselfly *Chalcopteryx rutilans* and obtained the variation of the relative permittivity across the nanolayers that compose the wing. Since there is a direct relation between the static relative permittivity and the refractive index in the visible range of the electromagnetic spectrum, we were able to reliably reproduce the spatial variation of the refractive index across the wing and therefore simulate its optical reflectance. In doing so, we showed that the vivid colors displayed by the *Chalcopteryx rutilans* wings are due to the periodic change in the refractive index across the wing, which is therefore shown to be a one-dimensional natural photonic crystal. The different colors seen in different regions of the wing are due to the number and thicknesses of the constituent nanolayers in each different color region. The refractive indexes in each color region change between approximately the same maximum and minimum values.

The direct relation between static relative permittivity and the visible refractive index means that the method demonstrated in this work is a reliable way of mapping the spatial profile of the refractive index of biological nanostructured systems.

## Experimental

### Wing sample preparation for EFM

Damselfly wing samples were produced by ultramicrotomy. Fragments of the chosen color region were embedded, without prior cleaning, in epoxy resin. The polymerized resin block was trimmed in a wedge shape, resulting in an orientation of the wing fragment perpendicular to the cutting plane. Sections 40 nm thick of the apex wedge were cut using a diamond knife and placed on 10 mm × 10 mm Au/Cr (60 nm/20 nm)-coated silicon wafer pieces. A conductive substrate surface is necessary for the proposed ε_r_ determination method. The sample also needs to be less than 100 nm thick to eliminate the influence of the stray capacitance [12]. Therefore, samples of three different color regions of the wing were prepared, namely blue, red and green/yellow ones.

An essential characteristic of the experiment is that both the sample and the conductive substrate need to be present within the AFM image. This is a key condition since the conductive substrate establishes a reference level in the analysis. Having both in the imaged region guarantees that the cantilever amplitude and, consequently, the effective radius of the tip will be the same for different materials, which is critical for the applied capacitance model [29]. Also, having the conductive surface and the sample in the same AFM image guarantees the precision in the determination of the thickness of the sample.

### Wing sample preparation for SEM

In order to study the layers of the wing a cross-sectional image of the fragment of the *Chalcopterix rutilans* male rear wing was obtained by SEM. Before preparing and polishing the cross section with a FIB, the wing was inserted in an evaporator to cover the surface with a thin Pt layer in order to make it conductive and reduce the curtain effect during FIB polishing. The Ga^+^ beam of the FIB was adjusted to 30 kV and 1 nA to mill a cross section of the wing while polishing was carried out under 30 kV, 16 kV and 5 kV, all of them with a beam current of 50 pA.

### Determination of the SPM parameters

The sample thickness *h* is directly determined via AFM imaging. The microscope control software also determines the resonance frequency *f*_0_ and the elastic constant *K* of the cantilever using the thermal tune method [30–31].

A critical parameter is the tip–sample distance *z*, that consists of the height *H*_lift_ plus the cantilever amplitude *Z*_amp_. The value of *H*_lift_ is adjusted and presented by the microscope with remarkably high accuracy and precision. The value of *Z*_amp_ is obtained using the standard amplitude–distance curve method.

Another critical parameter is the effective radius *R*. To obtain it, we carry out EFM measurements on a gold surface and determine the coefficient α. Over the gold surface, the effective thickness of the sample *h* goes to zero, thus *h*/ε_r_ = 0 [13,32], and Equation 6 can be seen as a function of *R*.

The coefficient α depends on *z* and to obtain the correct value of *R*, it is necessary to perform the EFM measurements of the sample and the substrate with the same height reference. EFM measurements with both substrate and sample in the same image solve this issue.

The conical angle is θ = 0.261 rad, as informed by the tip producer. To avoid the side effects of natural wear and contamination, different probes, with slightly different *K* values, were used for each region of the wing, in order to always have a new, clean probe in each measurement. We used the platinum-coated AC240TM-R3 probe model by Oxford Instruments Asylum Research in all measurements. The EFM mode used in this work is the standard one present in the Asylum Cypher ES SPM.

### Al_2_O_3_ reference sample

**Figure 9:**
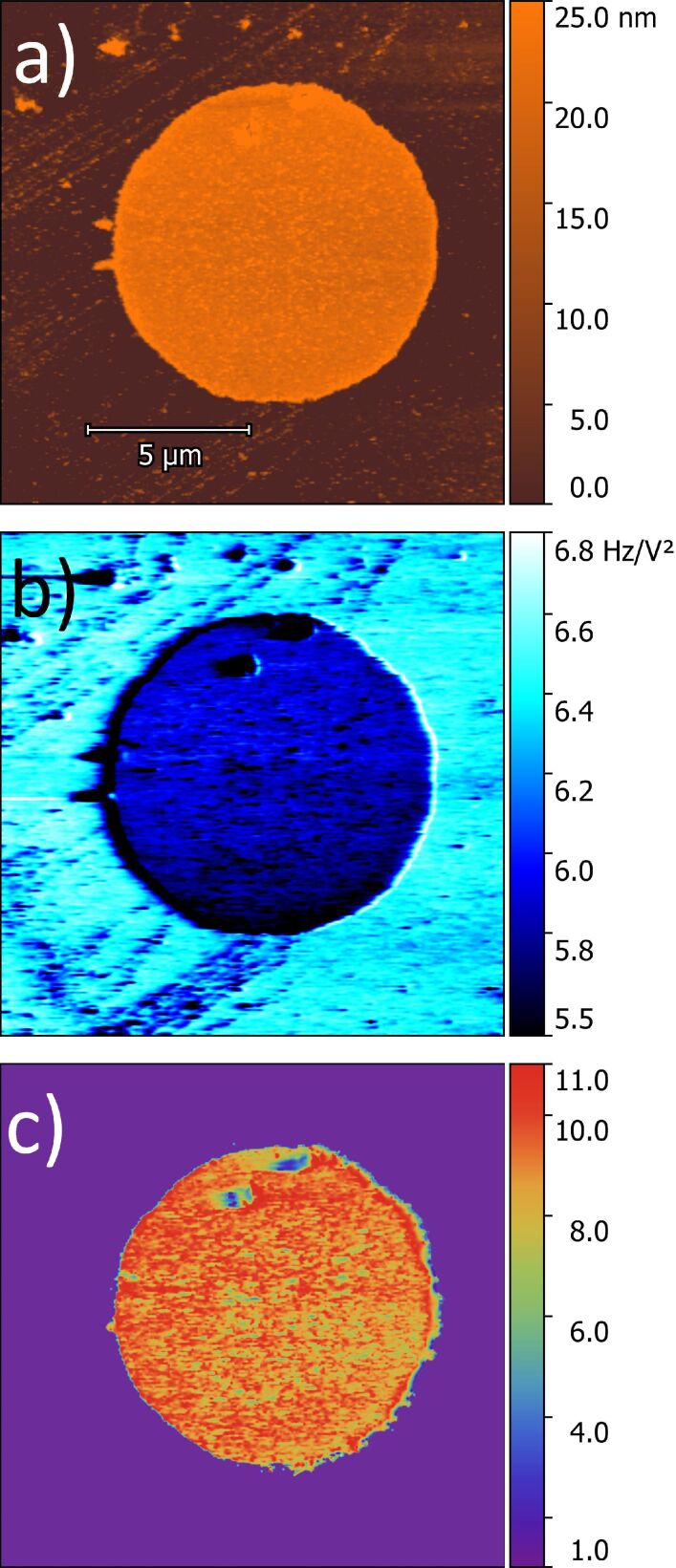
Thickness measurement of (a) the Al_2_O_3_ disk, (b) map of the α coefficient, and (c) dielectric constant map of the alumina film disk on gold.

We made reference samples of a material with a well-known relative permittivity. Applying our method to this reference sample, we validated the technique presented in this paper. Our reference samples were photolithographically defined disks of Al_2_O_3_ films with a radius of 5 µm deposited by ALD on Au/Cr (60 nm/20 nm)-coated silicon wafer pieces. The topographic image of an Al_2_O_3_ disk measured with AFM is shown in Figure 9a. The sample thickness was (21.0 ± 0.2) nm, relative to the gold surface.

In the EFM mode, the microscope measures the frequency shift for each bias voltage at each position on the sample. We varied the bias voltage from −10 V to +10 V, in steps of 1 V. Plotting the frequency shift as a function of the bias voltage, we obtain a parabolic function. Fitting the data with Equation 7 we obtain the α coefficient. Doing this operation for each pixel we build the image of α coefficients as we can see in Figure 9b. The gold surface has a higher α coefficient than the Al_2_O_3_ disk (Figure 9b). This means that the frequency shifts on gold are larger for all bias voltages, so the tip–substrate interaction on the gold surface is stronger than on the alumina disk. The tip radius was obtained using the α coefficient for measurements on the gold surface for *h* = 0, and was *R* = (36.7 ± 0.2) nm (Figure 9b). The tip–sample distance *z* is 44 nm, determined by the sum of 40 nm of the lift height of the setup and the 4 nm of the cantilever oscillation amplitude [16]. We choose a small cantilever oscillation amplitude during the EFM measurements in order to keep the tip close to the sample surface, in a range where this method is valid [12]. The free oscillating frequency *f*_0_ = 73.403 kHz and the cantilever elastic constant *k* = 2.24 N/m, were calculated using the thermal tune method.

The height *h* (Figure 9a) and the α coefficient (Figure 9b) are different for each pixel in the sample, the other parameters mentioned above and seen in Equation 6 are the same for all pixels. Solving Equation 6 for ε_r_ we constructed the map of the dielectric constant as shown in Figure 9c. In the reconstructed dielectric image, the region corresponding to the substrate was set to ε_r_ = 1 since this region corresponds to the relativity permittivity of air [15].

We obtained ε_r_(Al_2_O_3_) = 9.3 ± 0.2, which is in agreement with the values obtained by Yota et al., ε_r_ = 9.2 [33], and Biercuk et al., ε_r_ = 9 [34], both results being from Al_2_O_3_ produced by ALD, and also with the reference value for the dielectric constant of Al_2_O_3_ [35].

### Reflectance measurements

For measurements of the reflectance spectra, a halogen lamp with a color temperature of 3200 K (OLS1 FIBER ILLUMINATOR, Thorlabs) was used, which allows for reflection measurements in the spectral range from 350 to 1100 nm. A set of biconvex lenses focused the source light on the desired color region and another set of biconvex lenses delivered the reflected light to an optical fiber with 0.22 numerical aperture. The light was delivered through this optical fiber to the spectrometer (USB4000, Ocean Optics). Spectral data were acquired with the Spectra Suite software (Ocean Optics). Spectral measurements were made in three different color regions of male damselfly hind wings, namely yellow/green, red, and blue regions. In this work we study the light captured at 60° in relation to the normal of the wing.
